# Anti-Inflammatory and Anti-Allergic Properties of Colostrum from Mothers of Full-Term and Preterm Babies: The Importance of Maternal Lactation in the First Days

**DOI:** 10.3390/nu15194249

**Published:** 2023-10-02

**Authors:** Francesca Garofoli, Elisa Civardi, Camilla Pisoni, Micol Angelini, Stefano Ghirardello

**Affiliations:** Neonatal Unit and Neonatal Intensive Care Unit, Fondazione IRCCS Policlinico San Matteo, Piazzale Golgi 19, 27100 Pavia, Italy; lab.immunoneo@smatteo.pv.it (F.G.); c.pisoni@smatteo.pv.it (C.P.); m.angelini@smatteo.pv.it (M.A.); s.ghirardello@smatteo.pv.it (S.G.)

**Keywords:** colostrum, neonate, preterm newborn, anti-inflammatory components, anti-allergic components

## Abstract

Our narrative review focuses on colostrum components, particularly those that influence the neonatal immune system of newborns. Colostrum is secreted in small volumes by the alveolar cells of the breast during the first two to five days after birth. Colostrum is poor in fat and carbohydrates, with larger protein and bioactive compounds than mature milk. It plays a crucial role in driving neonates’ immunity, transferring those immunological factors which help the correct development of the neonatal immune system and support establishing a healthy gut microbiome. The newborn has an innate and adaptive immune system deficiency, with a consequent increase in infection susceptibility. In particular, neonates born prematurely have reduced immunological competencies due to an earlier break in the maternal trans-placenta transfer of bioactive components, such as maternal IgG antibodies. Moreover, during pregnancy, starting from the second trimester, maternal immune cells are conveyed to the fetus and persist in small quantities post-natal, whereby this transfer is known as microchimerism (MMc). Thus, preterm newborns are deficient in this maternal heritage, and have their own immune system under-developed, but colostrum can compensate for the lack. Early breastfeeding, which should be strongly encouraged in mothers of preterm and full-term babies, provides those immunomodulant compounds that can act as a support, allowing the newborn to face immune needs, including fronting infections and establishing tolerance. Moreover, making mothers aware that administering colostrum helps their infants in building a healthy immune system is beneficial to sustain them in the difficult post-partum period.

## 1. Introduction

Human milk (HM) is the first nutrient for newborns and extends the maternal immune influence into the post-natal period. Its composition, which includes nutrient and bioactive molecules, is dynamic. Its changes depend on the gestational age (GA) of the newborn at birth, stage of lactation, maternal diet and health, living environment, and other temporary factors. All these influences make maternal milk a tailored nutrient and a personalized immune treatment for the receiving infant [1,2]. We aim to describe the main immune characteristics of colostrum, the milk of the first 2–5 days postpartum that precedes transitional and mature milk (transitional/mature milk). Colostrum is a thick, yellowish-white fluid initially produced by the alveolar cells of the mammary gland during pregnancy that can be expressed during the third trimester and begins to be secreted after delivery [3]. Colostrum is poor in fat and carbohydrates but rich in proteins and with a larger amount of immune compounds than mature milk. Colostrum is a low-volume milk whereby around 2–10 mL of colostrum are expressed per feed [3,4,5]. Despite the small quantity, this first breast fluid is also mechanically advantageous to help the newborn to learn and regulate the suck, swallow, and breathe progression during feeding. Colostrum is the first colonizer of the offspring gastroenteric tract and is fundamental for the transfer of immune-active components with immediate effectiveness, but it also drives imprinting in founding a healthy and balanced neonatal immune system for full-term and preterm infants [3].

The newborn, especially if preterm, has an innate and adaptive immune system deficiency. In particular, phagocytosis, cell-mediated immunity, humoral immunity, and complement are undeveloped, lacking in efficiency and several bioactive components for exerting efficacious defense activities, resulting in increased newborn susceptibility to infection [3]. Early breastfeeding provides those immunomodulant compounds that can act as a support, allowing the newborn to face immune needs, including fronting infections and establishing tolerance [4,6]. Considering premature birth, the trans-placenta transfer of bioactive elements is precociously interrupted, as it happens for maternal IgG antibodies (IgGs), which cross, by transcytosis, the placental barrier reaching fetal circulation. Maternal IgGs represent the fetal dominant isotype and their levels are also correlated to neonatal protection against viruses and bacteria whose impact persists for decades after birth [6]. During pregnancy, maternal immune cells, identified as naive and memory T cells, B cells, NK cells, and monocytes, are also conveyed to the fetus and persist in small quantities post-natal for a variable period. This transfer is known as microchimerism (MMc). These maternal cells reach the fetus starting from the second trimester of gestation, and breastfeeding may favor persisting MMc higher levels during childhood. The mechanisms that support this persistence have yet to be fully investigated [6]. 

**Figure 1:**
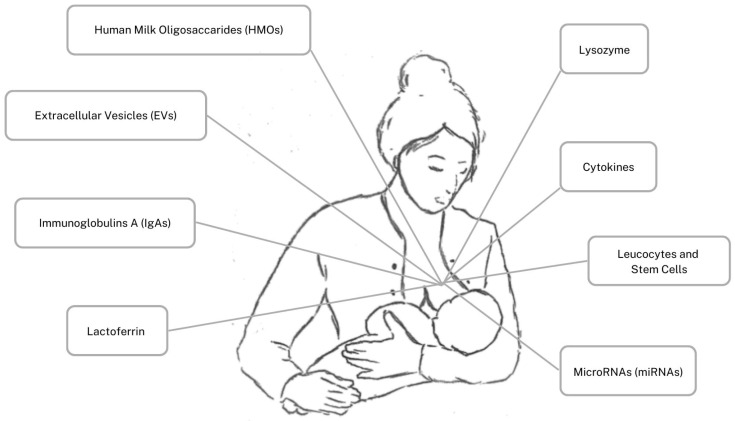
Human colostrum’s immune components.

Thus, preterm newborns are partially deficient of this maternal immune heritage, and do not have a fully developed immune system, but maternal colostrum may initially mitigate this deficiency by providing a high and active quantity of bioactive components. In comparison to transitional/mature milk, colostrum contains a higher concentration of Human Milk Oligosaccharides (HMOs), Extracellular Vesicles (EVs), secretory immunoglobulin A (IgA), Lactoferrin, lysozyme, cytokines, leucocytes, stem cells, and MicroRNA (miRNA. The immune components are illustrated in Figure 1 This peculiar composition supports both full-term and preterm newborns’ physiologically immature immune defenses, inhibiting infection, favoring immediate protection, laying the foundation for long-lasting immunity, and reducing the risk of inflammatory bowel diseases and allergies [1,2]. Noteworthy, in case of premature delivery, the composition of this fluid is even richer in immune components, as an extra protection for critical newborns who are more prone to infectious diseases and negative outcomes [5]. Hence, we aim to describe the colostrum most represented immune components, highlighting differences between full-term and preterm fluid and generally comparing to transitional/mature milk. We aim to highlight the essential immunomodulant properties of colostrum as the first offspring nutrient, essential in particular for preterm newborns, underlining the importance of maternal collaboration. Due to the fact that most of the premature newborns are not settled to be naturally breastfed, their mothers in particular are requested to make an effort to express their breast and collect colostrum to feed their neonate.

This goal can be reached with active support of the medical nursing staff for educating mothers that, beyond nutrition, the immune properties of colostrum can help strengthen the infant’s defense system.

## 2. Human Milk Oligosaccharides (HMOs)

Human Milk Oligosaccharides (HMOs), which play a role as bioactive substrates, are the third most represented components in colostrum and breast milk, after lactose (70 g/L) and lipids (40 g/L) [3,7]. HMOs are oligosaccharides containing a lactose-reducing end elongated with fucosylated and/or sialylated N-acetyllactosamine units, leading to over 200 HMOs. These soluble complexes are non-digestible sugars with various monosaccharides and chemical structures with similar characteristics and functions. They are determined by the α-1-2-fucosyltransferase (FUT-2) and d α-1-3-4-fucosyltransferase (FUT-3) genes, but other factors influence HMOs differences, such as maternal genetic, health, diet, preterm/full-term delivery, lactation period, geographical area, pollution, and other factors [8,9,10,11]. HMOs are prebiotics, which is to say a selectively fermented ingredient that results in specific changes in the composition, diversity, and/or activity of the gastrointestinal microbiota, thus conferring benefits upon host health [12].

HMOs have bifidogenic activity favoring the proliferation of specific Bifidobacteria (B), such as B infantis, B. breve, and B. bifidum, that overcome the growth of pathogenic microorganisms and decrease vulnerability to different diseases; specifically, they act against enteric diseases such as necrotizing enterocolitis (NEC) [11,12]. HMOs have been shown to directly or indirectly affect mucosal and systemic immunity, as well as to help reduce the risk of pathogenic infection, and they may support brain development [8,11,12]. They can modulate immune cells, pathogen recognition receptors, and signaling pathways related to the maturation of lymphoid tissue, thus influencing cytokine/chemokine networks suppressing pro-inflammatory compounds and regulating T helper 1/T helper 2 (Th1/Th2) lymphocyte balance [11,12]. Thus, they help prevent infection and support immediate and long-lasting immunity, reducing the risk of allergies, asthma, inflammatory bowel diseases, obesity, and metabolic disease [11,12]. HMOs concentrations are the highest in colostrum as 9–22 g/L [8] or 5 and 23 g/L [9], depending on the different environments (geographic area maternal health conditions, previous maternal pathogenic contacts, and other factors) and the reviewing methodology, followed by lower concentrations in transitional milk/mature milk, until 4–6 g/L after 6 months [8,9]. A study compared term and preterm colostrum and revealed no significant difference in the amount of HMOs between the two groups [13]. 

Due to their recognized immune benefits, HMOs have also been used to supplement infant’s formula to be more similar to human milk of the first days and to mature milk, depending on the nutritional composition of the formula and the prebiotic concentration. The results of different trials [7,8,11] indicated that supplementation in newborn’s formula favors an increase in gut Bifidobacteria, concurrently a decrease of pathogenic bacteria, reduced concentrations of plasma inflammatory cytokines, and a shift of T-cell responses to balanced T helper1 (Th1)1/T helper 2 (Th2) cytokine production. 

## 3. Extracellular Vesicles (EVs) 

Human colostrum and transitional/mature milk contain lipid bilayer-encased particles released from cells that send intercellular signals. Exosomes are the smallest size of these particles, named extracellular vesicles (EVs). EVs are produced by mammary epithelial cells, with a similar protein profile, or by local and remote cells passing through the blood or lymphatic circulation. Milk EVs carry countless proteins, lipids, HMOs, and non-coding RNAs, including miRNAs, immunoglobulins, lactoferrin, growth factors, hormones, and enzymes. 

The literature indicated that milk EVs are absorbed in the gut via endocytosis, acting locally or transported to distant organs, including brain, heart, liver, and spleen [14].

The richest exosome concentration is found in colostrum rather than in transitional/mature milk, reducing the risk of NEC in preterm infants [15,16,17]. The highest amount of EVs (1.62 × 108 particles/mL) is in colostrum from mothers to preterm infants which decreases after 2 weeks of lactation, reporting no association with maternal demographic or perinatal factors [15]. Also, HMOs packed in colostrum exosomes are more abundant and diverse than those in transitional/mature milk. It has been demonstrated that HMOs, delivered into macrophages via EVs, can stimulate phagocytosis, activate intestinal mucosa immunity, and attenuate Adherent-Invasive *E. coli* (AIEC) infection [16]. EVs can have anti-inflammatory, immunomodulatory, or neurodevelopmental effects. MiRNAs in EVs can alter immune cell gene expression, reducing viral transmission [14]. A study by Torregrosa Paredes et al. demonstrated that exosome phenotype in HM varies with maternal sensitization and lifestyle, which might influence allergy development in the future child [17]. Thus, EVs and their cargo seem to be very important in early post-natal life; they are highly represented in colostrum and present a variety of anti-inflammatory properties that deserve specific studies to determine the diversity of their immune benefits better.

## 4. Immunoglobulins A (IgAs)

Human colostrum and transitional/mature milk contain immunoglobulins, specifically high amounts of secretory immunoglobulins A (IgAs), the most represented immunoglobulin group. IgAs are antibodies active against all the pathogen microbes that host the maternal intestinal flora. The antigenic material resident in the mother’s gut (i.e., originating by microbes and food) is taken into the Peyer’s patches, creating multiple lymphocyte aggregates dedicated to the production of IgA dimers [18]. These cells migrate in the blood to mucosal membranes, including gut and exocrine glands. During late pregnancy, they ‘home’ to the mammary glands where they produce the IgA dimer antibodies with J chains. Thus, the complete antibody is secreted into milk and the result is more resistant to proteolysis than serum antibodies [18]. Since memory lymphocytes are brought into the lactating mammary glands, previous maternal antigenic experiences correspond to a broad set of various specificities of IgA antibodies in colostrum and milk [18]. It has demonstrated protection against various microbes, such as Vibrio cholerae, Campylobacter, Shigella, ETEC, and Giardia lamblia. IgAs have been demonstrated to protect infants against enteropathogens and necrotizing enterocolitis (NEC) and the activity can have multigenerational effects. These polymeric IgAs antibodies are more efficient in agglutination and virus-neutralization than monomeric antibodies [18]. IgAs can prevent the translocation of pathogenic bacteria across the epithelium and promote the establishment of symbionts in the gut through supporting microbial adherence to the epithelium and biofilm formation; they also mediate regulatory T cell homeostasis [17]. Thus, IgAs influence the developing gut microbiota by immediate pathogen exclusion and promote tolerance whereby the resulting healthy microbiota can influence the immune system continually [19]. The literature reported the highest range of IgA concentrations in colostrum (1.5 to 83.7 g/L), declining and fluctuating greatly in transitional/mature milk [19,20,21]. In particular, a review reported colostrum mean IgAs concentrations around 7.5 g/L and 1.6–2 g/L in transitional/mature milk, with wide variations among mothers and lactation period [20]. In this respect, Castellote et al. investigated IgAs concentrations in the colostrum of full-term (≥37 WE GA), preterm (30–37 WE GA), and very preterm infants (<32 weeks GA) and showed that the highest amount of IgAs was in preterm colostrum and the lowest amount was in very preterm colostrum [21]. These results highlight that the maternal lactogenic compensatory mechanisms, helping the development of preterm infant’s immunity, may activate after 30 weeks GA [21]. The same study showed that the colostrum of the preterm group was also extremely rich in bioactive factors, such as IL-6, TGFb1, and TGFb2. The interplay among IgAs, HMOs, and Lactoferrin may help reduce NEC risk due to intestinal immaturity and undeveloped immunity [15,22,23,24]. Metha et al. [25] evaluated IgAs, lactoferrin, and lysozyme concentrations in the HMs of mothers who delivered full-term or preterm infants (<32 GA); in both groups, milk of the first days (within day 8 post-partum) resulted in richer measures of these bioactive compounds than mature milk. IgAs were especially higher in preterm colostrum than in full-term colostrum. Thus, the high IgAs concentrations in maternal colostrum exerts dynamic protection with other bioactive elements, which is particularly needed by preterm infants. Nevertheless, particular attention has to be paid to exclusively breastfed infants of <30 weeks who demonstrate receiving less IgAs.

## 5. Lactoferrin

Lactoferrin is a key component of the mammalian innate response to infection produced by exocrine glands and released in high quantity in colostrum and maternal milk. Lactoferrin in human colostrum, and particularly in transitional/mature milk, has broad microbicide activity against gram-positive cocci, gram-negative bacilli, and fungi [22]. It plays a significant role in host-defense mechanisms through its iron-chelator, anti-inflammatory, antioxidant, and immunomodulatory properties. Indeed, Lactoferrin can be iron-free and chelate iron, avoiding bacterial growth or being saturated with iron, thus correcting iron deficiency. Due to antioxidant activities, Lactoferrin is an essential bioactive protein for preterm infants; it protects the developing brain from damage due to the excessive production of oxidative species and high levels of free iron [26] Pre-clinical evidence demonstrated that lactoferrin may counteract the potential influences of maternal nutrients able to alter milk composition thus hypothetically interfering with brain development. Moreover, protection of the preterm infant’s brain can start from maternal administration of lactoferrin, reducing birth-related pathologies, including preterm delivery, by decreasing the release of pro-inflammatory factors and inhibiting premature cervix maturation [26]. Lactoferrin’s highest concentration is in colostrum at up to 9 mg/mL and reduced to 1 mg/mL in mature milk, with no significant differences between preterm and full-term origin. Nevertheless, a trend has been observed for maintaining high protection in preterm colostrum to mature milk by supporting feeding premature infants with their mothers’ milk when their immune systems are most needed [27]. On the contrary, other data demonstrated higher lactoferrin levels in colostrum from full-term mothers rather than preterm mothers. [25]. Finally, two recent meta-analyses [28,29] considering studies on the role of oral lactoferrin supplementation in preventing sepsis and NEC concluded that this supplementation was associated with a reduction in late-onset sepsis in very and extremely preterm infants without decreasing the incidence of NEC stage II or III.

## 6. Lysozyme 

Lysozyme is another essential antimicrobial protein found in colostrum and HM. The enzyme, in association with lactoferrin, triggers platelet-activating factor and Interleukin-10 (IL-10), resulting in hydroxylation of the microbial b-glycosidic linkages and bacterial cell wall lysis. Lactoferrin can aid this procedure, acting in synergy [4]. Lysozyme is active against Gram (+) and Gram (−) bacteria. This protein kills gram (−) microorganisms. whereas Gram (+) microorganisms usually are inhibited in their growth.

Moreover, lysozyme promotes commensal bacteria growth [30,31]. As for IgA and lactoferrin, lysozyme is more represented in colostrum than in transitional/mature milk and preterm newborns are reported to have the highest concentrations able to support adaptive immunity and antibacterial activity. Higher lysozyme concentrations are related to the preterm group’s superior antibacterial and anti-inflammatory activities. Metha et al. [25] confirmed a greater amount of lysozyme in colostrum from preterm mothers than in that from full-term mothers. The relationship between maternal nutrition and lysozyme concentration in colostrum is not well defined [26,31]. 

## 7. Cytokines

Cytokines are signaling molecules synthesized by most of the nucleated cells and, once transferred via mother colostrum/milk, can cross the intestinal barrier and influence the infant’s immune system [32,33]. Ustundag et al. showed that colostrum contains higher cytokine concentrations than mature milk. In particular, the total amount of cytokines of colostrum from full-term mothers resulted in a larger amount than that from preterm mothers [32]. Specifically, interleukin (IL)-2 and tumor necrosis factor-alpha [TNF-α] levels were higher in full-term mothers’ colostrum, while IL-8 concentrations were similar in preterm and full-term groups. Conversely, considering IL-1β and IL-6 concentrations, the highest peak was reached in mature milk compared to colostrum [32]. 

Hrdý et al. described an allergic phenotype of colostrum composition from allergic mothers with a bias to a Th2-type response, characterized by an increased gene expression of IL-4 and IL-13 and decreased expression of Interferon-gamma (IFN-ϒ) in comparison to healthy mothers [33].

A large study [34] in the United Kingdom, Russia, and Italy found that cytokines, one of the major classes of immune components, are present in low concentrations and in some situations are even difficult to detect. Thus, the authors considered mothers from three different geographical regions, dissimilar environments, diet, and lifestyle, all factors that may potentially influence colostrum and HM composition, as well as neonatal health outcomes. The main results demonstrated quite homogenous findings. The presence of IL-2 and IL-13 were associated with less eczema incidence when detectable in colostrum and HM. Moreover, a large amount of IL-13 in colostrum had protective activity on food allergy and sensitization. In contrast, increased transforming growth factor β (TGF-β) 2 levels were associated with a higher incidence of reported eczema [34]. It is known that HM contains three different isoforms of TGF-β. Morita et al. found that eczema was associated with higher TGF-β1 levels in colostrum, but lower in 1-month milk, thus a lower TGF-β1 ratio (1-month milk/colostrum) was related to the development of eczema during the first 6 months of life, while the TGF-β2 ratio did not correlate to eczema later in life. Increased TGF-β1 levels may play a role in preventing allergic disease [35].

The recognized protective role of TGF-β is even more emphasized in a recent review by Gila-Diaz, [36], confirming that it reduces atopic sensitization by controlling the inflammatory processes. Furthermore, it has been reported that TGF-β could suppress neonatal T-lymphocytes activity to promote oral and intestinal tolerance. Finally, TGF-β stimulates the production of IgAs. 

Another anti-inflammatory cytokine is IL-10 whose concentration ranges from 5.9 to 7.3 ng/L in the colostrum of full-term mothers and from 1.1 to 8.8 ng/L in the colostrum of preterm mothers. Differences in IL-10 concentrations between preterm and full-term milk are still controversial, but it has been reported that lower IL-10 levels are related to an increased risk of NEC [36]. Conversely, pro-inflammatory cytokines, such as tumor necrosis factor-alpha (TNFα), IL-1, IL-6, IL-8, and IFN-ϒ are present at low concentrations, depending on GA [36]. For example, IL-6 concentrations vary between 4.4–340 ng/L in full-term mothers’ colostrum, between 15.3 -362 ng/L in preterm, and between 9.3–67.9 ng/L in very preterm. The same trend has been found for IL-8 whereby concentrations were 0.04–26.3 μg/L in full-term, 0.13–14.7 μg/L in preterm, and 0.1–3.0 μg/L in very preterm colostrum [36]. Nevertheless, a further study demonstrated the highest levels of IL-8 in colostrum with a decline with the lactation stage. However, no differences were observed in its concentration between full-term and preterm colostrum [37]. 

## 8. Leucocytes and Stem Cells

Leukocytes in colostrum and transitional/mature milk include granulocytes and mononuclear leukocytes (lymphocytes, monocytes, and macrophages), mammary epithelial cells, and stem cells, in a variable concentration of 10^2^–10^5^ cells/mL, depending on the stage of lactation and mother/offspring health status, on their infection status, and on individual variation [38,39]. Leukocytes have a critical defensive role for the neonate and increase during maternal and infant infection. The leukocyte population comprises B and T cells. In particular, of the viable leukocytes, 5%–10% are T cells. [6,38,39]; Immunocytological T lymphocyte detection demonstrated that CD3+ accounted for 57.8 ± 4.2%, CD4+ accounted for 31.5 ± 3.7%, CD8+ accounted for 26.2 ± 2.4%, and CD4+/CD8+ accounted for 1.2 ± 0.3 in colostrum [39]. CD3+, CD8+, and C4+ concentrations are significantly higher in colostrum than in transitional milk/mature milk and vary depending on maternal conditions, socioeconomic status, and availability to primary healthcare services. [38,39,40]. An increase in CD4+ regulatory T (T reg) cells, together with other bioactive components, such as HMOs and IL10, is reported with the development of tolerance, thus decreasing the risk of food allergy [41]. 

Trend et al. [42] highlighted that the CD45+ cell concentration was higher in colostrum than in mature milk, decreasing with the progression of lactation, and in particular they observed no differences between preterm and full-term milk in leukocyte concentration, though minor differences occurred in some leukocyte frequencies. Thus, this study suggests that the increased risk of infection in preterm infants is not associated with deficiencies in leukocyte concentration/activity from breastfeeding [42]. Wu et al. [1] studied dynamic changes in cellular components in colostrum and mature milk and showed different proportions with the stage of lactation of hematopoietic stem cells (CD34+, CD117+, and CD133+), hematopoietic cells (CD45+), and mesenchymal stem cells (MSCs) (CD90+, CD73+, CD44+, CD105+, CD271+, and CD146+). The authors correlated the amounts of various cellular components of HM at 3 and 6 months of baby’s age, considering maternal history of allergy, feeding patterns, and infantile disease. This first study on the clinical impacts of stem cells on infantile diseases demonstrated that a high percentage of CD34+ cells in colostrum, as well as a high percentage of CD133+ cells and a low percentage of CD105+ cells in mature milk, was associated with a significantly increased risk of infantile eczema within their first 3 months after birth [1]. Previous reports [43] showed that blood CD34+ could promote allergic inflammation and eczema is an inflammatory disease. Other authors [44] characterized different cell subsets in human colostrum and transitional milk and identified the proportion of these cell subsets. They used hematopoietic stem and progenitor cell markers as CD45, as follows: CD45dim/+ (hematopoietic stem/progenitor cells) and CD45- (non-hematopoietic stem/progenitor cells). The findings indicated that the percentage of CD45-CD34+ cells was predominant in both colostrum and transitional milk. The rate of CD45+/CD133+ cells was high in colostrum, while the percentage of CD45-CD133+ cells was high in milk. The results showed that early milk is an abundant reservoir of hematopoietic stem/progenitor-like cells in the CD45 ^+high^ population and non-hematopoietic stem/progenitor-like cells in the CD45- population. Nevertheless, the lack of a consensus protocol for characterizing different phenotypes of stem/progenitor cells in HM does not allow us to standardize a univocal method to measure and typify HM phenotypes of this cell population. Thus, it is still a matter of debate regarding the significance and impact of stem/progenitor cells on the recipient neonate.

## 9. MicroRNAs (miRNAs)

Among the immune bio-component of human colostrum, we can account for microRNAs (miRNAs), small, non-coding fragments of RNA able to regulate gene expression by post-transcriptional changes of mRNA strands. They originate in maternal mammary epithelial and immune cells or other tissues reaching breast milk through blood circulation [45]. MiRNAs are mainly packed into EVs which macrophages can collect and transfer from the mother to the infant. Indeed, they are involved in several pathways that influence oral tolerance development [45]. Many different types of miRNAs have been detected in colostrum and HM with potential biological implications for cell communication, fatty acid biosynthesis, immune pathways, developmental timing, and metabolism because they can modulate the activity of specific mRNA targets [46]. In particular, a trial about the expression of adipogenesis-related miRNAs (let-7a, miRNA-30B, and miRNA-378) indicated that these were expressed in colostrum and mature milk and were related to maternal weight and infant gender. The concentrations of miRNA-30B, let-7a, and miRNA-378 in colostrum were negatively correlated with maternal pre-pregnancy BMI and, in mature milk, let-7a was negatively correlated with maternal weight late in the pregnancy. Moreover, miRNA-30B and miRNA-378 resulted in greater amounts in the colostrum received by girls than in that received by boys. Thus, this result highlights a possible mechanism by which obesity susceptibility can be transferred from the parents to the next generation [46]. Thus, the trial demonstrated that the expression of adipogenesis-related miRNAs is associated with maternal factors and can be affected by a high-fat diet, chronic hyperglycemia, obesity, and inflammation. The authors concluded that the influencing factors of miRNAs and their biological functions in milk are far from being well studied [46].

Kosaka et al. [47] first observed a high content of miR-181 and miR-155 in colostrum and milk, apparently involved in B cell differentiation, possibly facilitating T reg cell development. Other primarily represented miRNAs, such as miR-17 and miR-92, have been associated with the regulation of monocyte development and the differentiation and maturation of B and T cells. Lastly, miR-223 has been associated with the proliferation of granulocytes [47]. 

A recent Mexican investigation that aimed to compare the expression of five immune-regulatory miRNAs in HM samples of full-term mothers with formula concluded that the expression of the immunomodulatory miRNAs was similar in all the human colostrum and mature milk samples and was higher than in formula milk [48]. MiRNAs are nowadays deeper studied since they are associated with immune system compounds, including TGF-β, T cell receptor, Toll-like receptor signaling, Janus kinase-signal transducer and activator of transcription (JAK-STAT), and Th1 and Th2 cells differentiation. Nevertheless, some of the most well-known miRNAs, having essential and well-described immune functions, are expressed in low amounts and are not among the most expressed miRNAs [45]. For example, Hicks et al. [49] demonstrated that miR-375 promotes the differentiation of T reg cells during allergic rhinitis, decreasing the risk of atopies, such as atopic dermatitis, food allergies, or wheezing.

Zeng et al. [50] showed that miR-181a and miR-155 were correlated with the proliferation and function of T-reg cells in allergic rhinitis. Specifically, miR-155 promotes the differentiation of T reg cells, whereas miR-181a regulates the expression of IL-10 and TGF-β in allergic rhinitis. The authors (unpublished data) found that allergic children had decreased T reg cell number and function. 

## 10. Discussion

The newborn, especially if preterm, has an underdeveloped innate and adaptive immune system with a consequent increase in susceptibility to infection [4,5]. Early breastfeeding provides those immunomodulant compounds which can act as a support at birth for preventing immediate risks of infections and diseases, while driving towards improving the off-spring’s immune system and establishing tolerance with a decreasing risk of developing allergic diseases [2,3,4,5,6]. 

This review highlights how most of the immune components, particularly anti-inflammatory and anti-allergic elements, are more represented in colostrum than in transitional/mature milk. The reason can be that, in the very first days of life, the newborn has to face the distressing and sudden passage from fetal to post-natal life and the larger amount of immune compounds from colostrum is extremely needed to support the off-spring’s immature defense system. Table 1 summarizes immune components of colostrum from preterm, term mothers and transitional/mature milk; Table 2 reports the main functions of these components in colostrum. 

Maternal colostrum dynamically changes its immune structure into mature milk while the neonate is growing and simultaneously increasing its immunity level. The immune composition of colostrum evolves not only with the neonate’s age progression, but also depending on whether the infant is born full-term or preterm. Preterm newborns are even more prone to undergo infectious disease having a particular blunted immune system, so maternal colostrum balances with a larger quantity of immune components than full-term colostrum. [20,21,22,23,25,32]. Moreover, new evidence demonstrated that oral colostrum priming improves immune protection and reduces the time to full enteral feeding in preterm infants [5,51,52]. Nevertheless, difficulties in nourishing with colostrum, which has an inverse rate between richness of immune components and volume, are extremely common. In general, these mainly depend on maternal education with family and hospital staff support during the very first day after delivery. Mothers have to be motivated about the importance of these factors in the decision-making process for breastfeeding. They have to be informed that colostrum is the main first dietary intervention for a competent immune system presenting extra value for a neonate’s growth in critical situations like a preterm birth. For mothers of preterm neonates, the possibility to give their own colostrum is an incentive that makes them conscious of its importance in the growing and recovering process of their neonate. When preterm infants are unable to be breastfed or in case of maternal breast problems, the practice of hand expression or pump expression is used even though it can be tiring and unsatisfying. Making mothers aware that colostrum facilitates their infants in building a healthy immune system that is able to fight disease is an approach that can be useful to support these women in the post-partum period.

## 11. Conclusions

The presence and efficacy of many specific immune system nutrients in colostrum, tailored to newborn’s needs, can balance the immature defense system of the offspring and empower mother accountability. Thus, we can conclude that colostrum can comply with the definition of “immuno-nutritherapy” [22,31]. 

## Figures and Tables

**Table 1:** Different concentrations of the immune components through lactation period: a semi- quantitative comparison among preterm colostrum, full-term colostrum, and mature milk.

Immune Component	Colostrum Concentration (to Preterm Mothers < 37 WE GA)	Colostrum Concentration (to Full-Term Mothers > 37 WE GA)	Transitional/Mature Milk Concentration
HMOs	+ + +	+ + +	+
EVs	+ + +	+ +	+
IgAs	+ + + (- very preterm < 30WE GA)	+ +	+
Lactoferrin	+ + +	+ + +	+
Lysozyme	+ + +	+ + +	+
Cytokines (considered as total)	+ +	+ + +	+
Leucocytes and Stem cells	+ + +	+ + +	+
MicroRNAs	+ + +	+ + +	+ + +

**Table 2:** Summary of the main functions of immune components in colostrum.

Immune Components	Main Immune Activity *
HMOs	Prebiotic with bifidogenic activity, prevent infection and support immunity, reducing the risk of allergies, asthma, inflammatory bowel diseases, obesity, and metabolic disease
EVs	EVs and their cargo present a variety of anti-inflammatory properties
IgAs	Antibodies protecting against various microbes prevent the translocation of pathogenic bacteria across epithelium and promote establishment of symbionts in the gut and biofilm formation, mediate regulatory T cell homeostasis
Lactoferrin	Iron-chelator, with anti-inflammatory, antioxidant, immunomodulant properties. Protect the developing brain from excessive production of oxidative species
Lysozyme	Enzyme active against bacteria, kills gram (−) microorganisms Gram (+) are inhibited in their growth, promotes commensal bacteria growth
Cytokines (considered as total)	Signaling molecules with anti or pro inflammatory action. Regulate allergic disease, including atopy and food allergy
Leucocytes and Stem cells	Development of tolerance, decreasing risk of allergy On the opposite, in different proportion can promote allergic inflammation and eczema. Reservoir of hematopoietic stem/progenitor-like cells and non-hematopoietic stem/progenitor-like cells
MicroRNAs	Regulation of monocyte development differentiation and maturation of B, T cells and granulocytes. They are involved in allergic rhinitis

* References Are Reported in the Relevant Section of the Text.

## Data Availability

No new data was created or analyzed in this study. Data sharing is not applicable to this article.
